# Organ Mass Variation in a Toad Headed Lizard *Phrynocephalus vlangalii* in Response to Hypoxia and Low Temperature in the Qinghai-Tibet Plateau, China

**DOI:** 10.1371/journal.pone.0162572

**Published:** 2016-09-07

**Authors:** Jimin Han, Ronghui Guo, Jiaqi Li, Chen Guan, Yu Chen, Wei Zhao

**Affiliations:** 1 School of life sciences, Lanzhou University, Lanzhou, Gansu province, China; 2 Nanjing Institute of Environmental Sciences, Ministry of Environmental Protection, Nanjing, Jiangsu province, China; Sichuan University, CHINA

## Abstract

Hypoxia and low temperature at high altitudes are the main environmental pressures for alpine animals, inducing phenotypic plasticity at several levels. To investigate the effect of these variables on the organ mass of *Phrynocephalus vlangalii*, 138 individuals belonging to four populations living along an altitudinal gradient in the Qinghai-Tibet Plateau (China) were dissected to remove heart, lungs, stomach, and intestinal tract. Organ dry mass, individuals’ sex, and body mass, as well as mean annual temperature and average air pressure (calculated from a 30-year-data series obtained from the National Climatic Data Center) were subjected to two-way analyses of covariance and generalized linear mixed models (GLMMs). Except for the heart, organ mass varied significantly among populations, although only lung and stomach mass increased significantly with increasing altitude. Males’ heart and lung mass was higher than that of females, which might be due to their different behavior and reproductive efforts. GLMM analyses indicated that air pressure had a positive effect on heart, lung and intestinal tract mass, whereas temperature had a negative effect on these three organs. In order to explain the effect of hypoxia and low temperature on *P*. *vlangalii*’s organ mass, further rigorous study on respiration, energy budget and food intake was encouraged.

## Introduction

Phenotypic plasticity, the ability of a genotype to produce different phenotypes across environmental conditions, is a tactic enabling organisms to adapt to heterogeneous environments [1,2]. This plasticity might be induced by short-term acclimatization or long-term evolutionary adaptation, involving phenotypic components such as morphology and physiology [3–6]. To some extent, classic ecological rules might be regarded as phenotypic plasticity descriptors along an environmental gradient. High altitude, associated with low temperature and hypoxia, has attracted much attention from evolutionary biologists and physiologists [5,7,8], as it is a particularly severe challenge to animals triggering a series of phenotypic variations.

In terms of physiological changes, high altitude exposure alters cardiovascular functions such as cardiac output, heart rate, and blood pressure [9,10], and hemodynamics, including hemoglobin content and blood-O_2_ affinity [5,6]. Organ mass also changes as altitude increases, and an increase in heart mass due to high altitude exposure has been reported [4,6]. However, many of the known biological responses to hypoxia were not derived from studies based on plateau native species, and thus the phenotypic plasticity induced by hypoxia exposure referred therein might be the result of maladaptation (reviewed in [5]). Moreover, most studies conducted so far were based on endotherms, with only a few recent researches focusing the physiological adaptation to high altitude in ectotherms [6,11,12]. Yet, organ mass variation with altitude increase remains largely unknown.

The Qinghai-Tibet Plateau, with more than one million km^2^ and an average elevation exceeding 5000 m, is the largest and highest plateau on Earth. The uplift of the plateau remodeled the geomorphology of China and changed the climate in East Asia [13]. A series of orogenic events affected the evolutionary processes of the species inhabiting this area [14–16], leading to the formation of endemic species such as *Phrynocephalus vlangalii*. This small lizard lives in altitudes ranging from 2300 to 4500 m [17], being the ideal model for studying phenotypic plasticity in high altitude. The present research aimed to (1) examine the phenotypic plasticity of *P*. *vlangalii* organs along a natural altitudinal gradient, verifying if the phenotypic plasticity in this ectotherm follows the typical rules observed in small mammals (endotherms), and (2) investigate the effects of low temperature and hypoxia on *P*. *vlangalii* organ mass. We hypothesized that populations living at high altitude would have larger heart and lung masses, because of low oxygen pressure, while the mass of digestive organs would remain unchanged, due to *P*. *vlangalii*´s ectothermic and low energy-expenditure.

## Materials and Methods

Our experimental procedures complied with the current laws on animal welfare and research in China and were specifically approved by the Animal Research Ethics Committee of Lanzhou University.

**Fig 1:**
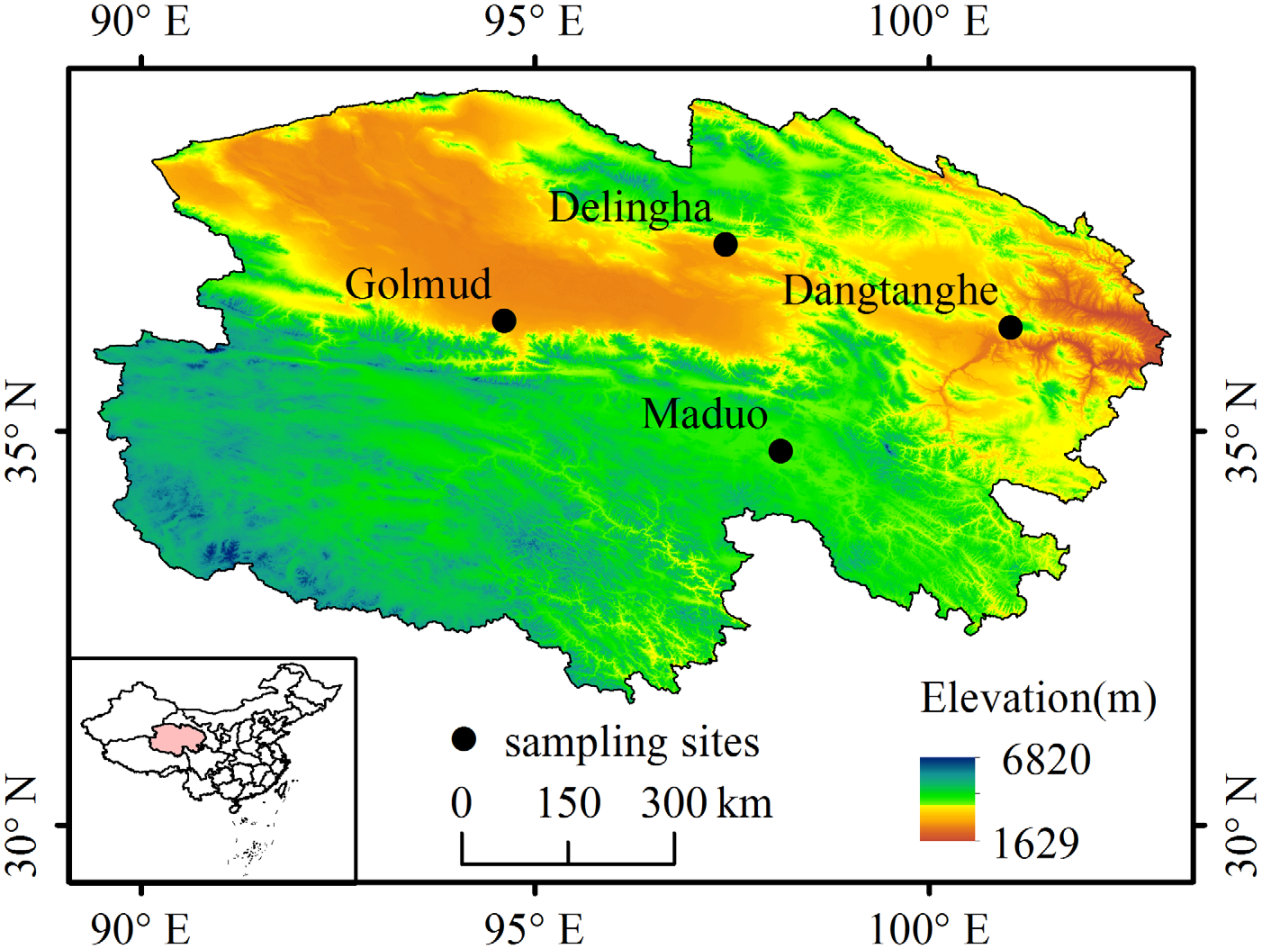
*Phrynocephalus vlangalii* sampling sites. The four populations of *P*. *vlangalii* were sampled in the displayed locations along the Qinghai-Tibet Plateau, China. The base map was downloaded from Geospatial Data Cloud.

One hundred and thirty eight *P*. *vlangalii* individuals, stored in absolute ethanol, were obtained from Room 419, School of life Sciences, Lanzhou University (the sample ID see S1 Table). These individuals were collected in four locations along an altitudinal gradient (2810 to 4250 m) in the Qinghai-Tibet Plateau (Fig 1) in summer 2010, 2011, and 2012. Only adult individuals whose snout-vent length (SVL) was larger than the smallest size at maturity were considered in this study. The size at sexual maturity for each population was based on the SVL of the smallest *P*. *vlangalii* females containing embryos within that population [18], as the size at sexual maturity varied with altitude. To avoid measurement errors, the SVL and body mass of each individual considered in this study were those registered in the specimen label. Some individuals only allowed collecting data for two organs, because they were used in other research (Table 1). As the specimens were storage in absolute alcohol and the lipids in organs might be dissolved, readers must take care in comparing our result with other studies.

**Table 1:** Altitude and climatic factors in the four sampling locations of *Phrynocephalus vlangalii* within the Qinghai-Tibet Plateau (China) and the several phenotypic variables considered in the present study.

Population	Sex	N	SVL (mm)	Body mass (g)	Heart mass (mg)	Lung mass (mg)	Stomach mass (mg)	Intestinal tract mass (mg)	Altitude (m)	Temperature(°C)^a^	Air pressure (hPa) ^a^
Golmud	**Males**	12	55.38±1.55	6.01±0.47	8.1±0.8	7.6±0.6	13.2±1.7	11.1±1.1	2810	5.8	724.7
	**Females**	7	53.04±1.28	5.60±0.39	5.5±0.6	5.8±0.6	11.0±1.0	11.3±0.8			
Delingha	**Males**	18	54.90±1.00	6.12±0.33	7.9±0.7 (17)^b^	7.8±0.7 (17)	10.7±1.0 (14)	17.1±1.7 (14)	2900	4.4	708.7
	**Females**	28	55.22±0.89	8.25±0.40	9.8±0.9	8.6±0.5	16.1±1.7 (14)	19.8±1.7 (14)			
Daotanghe	**Males**	13	54.81±0.57	5.74±0.22	10.5±1.0 (11)	10.8±0.6 (11)	17.1±2.6 (4)	20.3±3.1 (4)	3367	0.8	688.8
	**Females**	18	54.37±0.63	6.69±0.36	7.6±0.5	7.7±0.6	15.5±2.7 (6)	20.6±1.9 (6)			
Maduo	**Males**	19	56.05±0.47	7.31±0.31	10.2±0.8 (13)	11.4±0.5 (13)	12.4±1.2	18.8±1.5	4250	-3.3	604.3
	**Females**	23	56.71±0.68	8.59±0.44	8.4±0.7	9.5±0.5	14.5±1.3 (15)	23.6±3.0 (15)			

SVL, snout to vent length.

^a^ average values based on a 30-year-data series obtained from the National Climatic Data Center, China.

^b^ numbers between brackets indicate sample size; some specimen’s preservation status only allowed collecting data for two organs

Adult specimens were dissected to remove the heart, lung, stomach, and intestinal tract. After removing fat and connective tissue, organs were dried for 72 h at 60°C and weighed to obtain their dry mass, which was used in the subsequent analysis. To evaluate the effect of environment on organ mass, mean annual temperature and average air pressure were calculated based on a 30-year-data series obtained from the National Climatic Data Center (China).

All morphological data were log-transformed to eliminate the effect of index dimension and meet the required normality and homogeneity assumptions. Then a two-way analysis of covariance (ANCOVA) was used to test differences in organ mass among the four populations. Organ mass was the dependent variable, population and sex were the fixed factors, and body mass was the covariable. Unstandardized residuals from the regression of organ mass on body mass were calculated to produce size-adjusted variables. After removing the effect of body mass, a linear regression analysis between organ mass and altitude was conducted for the entire population and for males and females, separately. Finally, generalized linear mixed models (GLMMs) were used to test the effect of climate on organ mass (dependent variable), setting mean annual temperature and air pressure as the fixed effect, and population, sex, and body mass as random factors. Statistical significance was evaluated using Type III sums of squares tests in IBM SPSS 20 (IBM Corp., New York, USA), considering *P* < 0.05 as the significance level.

## Results

Data obtained for the 138 adult specimens are listed in Table 1. 53 individuals only allowed collecting heart and lung masses, and three individuals only collecting stomach and intestinal tract masses.

**Fig 2:**
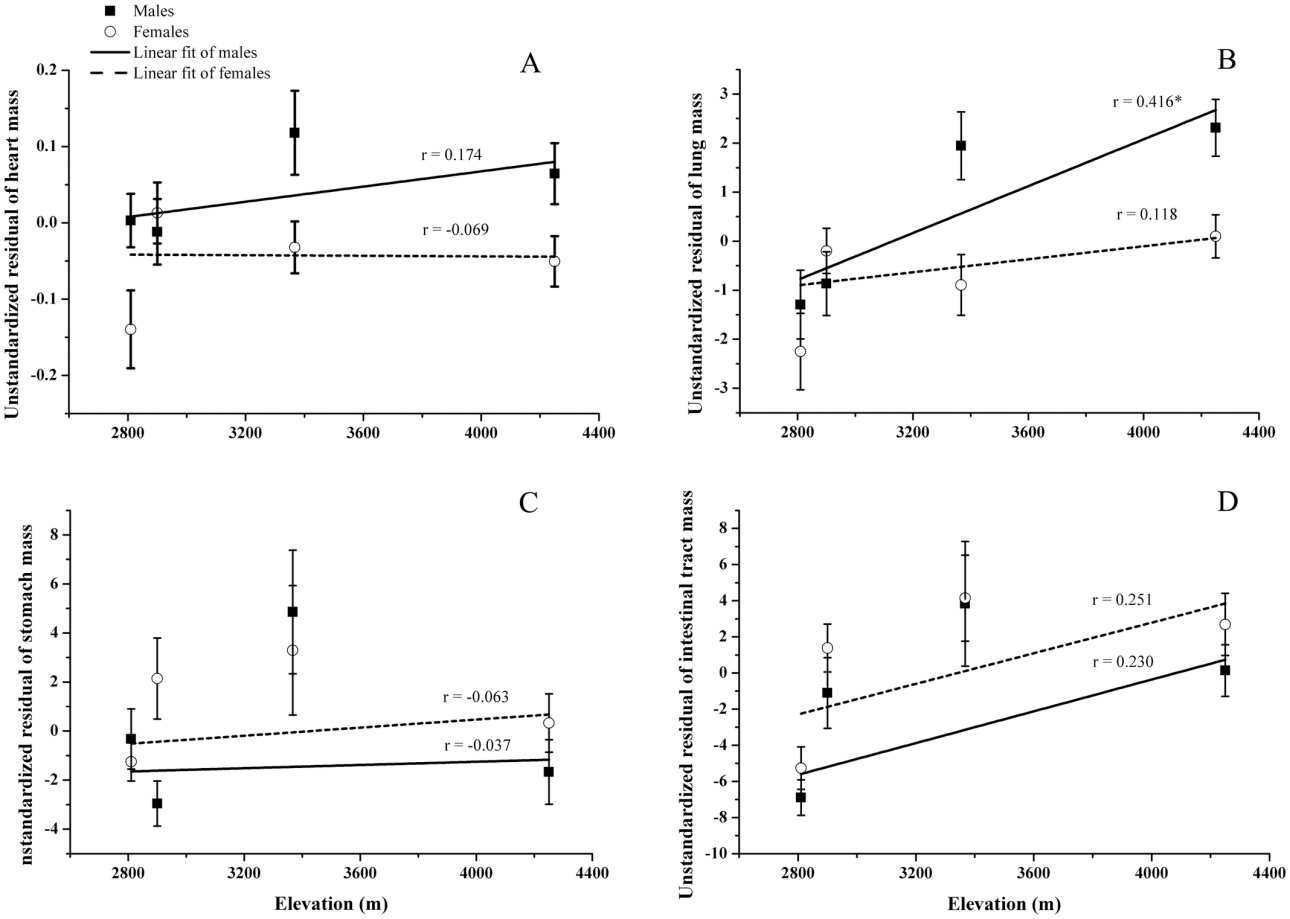
Altitudinal variation of *Phrynocephalus vlangalii* organ mass in the Qinghai-Tibet Plateau, China. (A) heart, (B) lung, (C) stomach, (D) intestinal tract. The effect of body mass was eliminated using the unstandardized residuals obtained from the linear regression analysis performed for each organ. Black squares and empty circles represent the means for males and females, respectively, and the error bar represents the standard error. The solid and dotted lines represent the fitting of organ mass to altitude for males and females, respectively. The coefficients of the linear regression analysis are displayed above lines and the asterisk (*) denotes *P* < 0.05.

Two-way ANCOVA results indicated that, after controlling for body mass, sex had a significant effect on heart and lung mass, but not on stomach and intestinal tract mass (Table 2). In the four populations studied, males had relatively larger heart and lung mass than females, but similar stomach and intestinal tract mass (Fig 2). The mass of lung, stomach and intestinal tract was differed significantly among populations, while the mass of heart was similar among populations (Table 2). None of the interactions between sex and altitude was significant (Table 2).

**Table 2:** Effects of altitude and sex on organ mass in *Phrynocephalus vlangalii*.

Organ	Males vs. females	Altitude	Interaction^a^
Heart	**F**_**1,126**_ = 8.426, P* **= 0.004**	F_3,126_ = 1.188, *P* = 0.317	F_3,126_ = 2.310, *P* = 0.080
Lung	**F**_**1,126**_ **= 19.911, *P* < 0.001**	**F**_**3,126**_ **= 5.970, *P* = 0.001**	F_3,126_ = 2.579, *P* = 0.057
Stomach	F_1,76_ = 0.012, *P* = 0.914	**F**_**3,76**_ **= 2.946, *P* = 0.038**	F_3,76_ = 1.293, *P* = 0.283
Intestinal tract	F_1,76_ = 0.346, *P* = 0.558	**F**_**3,76**_ **= 5.745, *P* = 0.001**	F_3,76_ = 0.445, *P* = 0.721

^a^ Data were analyzed by two-way ANCOVA using body mass as covariable.

* Values are significant at *P* < 0.05.

Linear regression analysis showed that only lung and intestinal tract mass increased significantly with increasing altitude (Table 3, Fig 2). When males and females were analyzed separately, only the lung mass of males increased significantly with increasing altitude (Table 3).

**Table 3:** Relationship between organ mass and altitude, based on linear regression analyses.

Organ	Males	Females	Total
Heart	r^a^ = 0.198, F_1,57_ = 2.317, P* = 0.134	r = -0.116, F_1,74_ = 1.001, *P* = 0.320	r = 0.019, F_1,133_ = 0.047, *P* = 0.828
Lung	**r = 0.448, F**_**1,57**_ **= 14.342, *P* < 0.001**	r = 0.159, F_1,74_ = 1.931, *P* = 0.169	**r = 0.283, F**_**1,133**_ **= 11.550, *P* = 0.001**
Stomach	r = -0.050, F_1,41_ = 0.101, *P* = 0.753	r = -0.067, F_1,40_ = 0.181, *P* = 0.673	r = 0.046, F_1,83_ = 0.174, *P* = 0.678
Intestinal tract	r = 0.186, F_1,41_ = 1.474, *P* = 0.232	r = 0.277, F_1,40_ = 3.322, *P* = 0.076	**r = 0.244, F**_**1,83**_ **= 5.267, *P* = 0.024**

^a^ The effect of body mass was eliminated using the unstandardized residuals obtained from the prior linear regression analyses of organ mass on body mass.

* Values are significant at *P* < 0.05.

As indicated by the GLMM coefficients presented in Table 4, which represent the slope of the relationship between organ mass and each predictor variable, partial pressure of oxygen (air pressure) had a significant and positive effect on heart, lung and intestinal track mass, but not on stomach mass. Temperature had a significant negative effect on heart, lung and intestinal track mass, but not on stomach mass.

**Table 4:** Influence of low temperature and hypoxia on *Phrynocephalus vlangalii* organ mass, based on generalized linear mixed models.

Source of variation	Random effect				Fixed effect			
	Var	S.E.	Z-value	P-value*	Coefficient	S.E.	t-value	P-value*
**Heart mass**								
Residual	0.029	0.004	7.693	**<0.001**				
Population	0.001	0.059	0.017	0.987				
Sex	0.004	0.004	0.926	0.354				
Body mass	0.255	0.264	0.965	0.335				
Intercept					-15.777	6.102	-2.585	**0.011**
Air pressure					0.025	0.009	2.688	**0.008**
Temperature					-0.316	0.122	-2.577	**0.011**
**Lung mass**								
Residual	0.013	0.002	7.859	**<0.001**				
Population	0.000 ^a^							
Sex	0.004	0.003	1.156	0.247				
Body mass	0.249	0.198	1.263	0.207				
Intercept					-8.292	4.427	-1.873	0.063
Air pressure					0.014	0.007	1.999	**0.048**
Temperature					-0.186	0.089	-2.099	**0.038**
**Stomach mass**								
Residual	0.020	0.003	6.123	**<0.001**				
Population	0.000 ^a^							
Sex	0.000	0.001	0.216	0.829				
Body mass	0.623	0.504	1.236	0.217				
Intercept					-10.856	7.442	-1.459	0.148
Air pressure					0.018	0.011	1.546	0.126
Temperature					-0.290	0.152	-1.917	0.059
**Intestinal track mass**								
Residual	0.021	0.003	6.251	**<0.001**				
Population	0.000 ^a^							
Sex	0.000 ^a^							
Body mass	0.332	0.288	1.152	0.249				
Intercept					-14.989	6.786	-2.209	**0.030**
Air pressure					0.024	0.010	2.327	**0.022**
Temperature					-0.313	0.138	-2.272	**0.026**

^a^ This parameter was redundant.

* Values are significant at *P* < 0.05.

## Discussion

Our results showed that, with the exception of heart mass, organ mass changed with increasing altitude, although only lung and intestinal track mass increased significantly with increasing altitude. There was no significant correlation between altitude and heart or stomach mass. Sex only showed a significant effect on heart and lung mass. The main effect of hypoxia was the decrease in heart, lung and intestinal track mass, while low temperature increased the mass of heart, lung and intestinal track mass.

The effect of sex might be due to the differences in reproductive role and behavior between the two sexes. Under sexual selection, *P*. *vlangalii* males usually maintain a larger territory than females during reproduction [19,20]. As a result, males not only frequently show mating behaviors, but are also more territorial [21]. These performances require expending vast amounts of energy, so sexual selection might have indirectly affected males’ cardiorespiratory system, leading to the evolution of more powerful heart and lungs in males than in females in order to provide more energy to muscle cells.

Vertebrates living at high altitude are not able to avoid hypoxia exposure only by changing their behavior. Many endotherms can compensate hypoxia by reducing O_2_ demand through metabolism suppression [22] and ectotherms mainly adapt to hypoxia through metabolic depression [23,24]. Alternatively, several physiological adjustments might be performed to preserve O_2_ supply in hypoxia [5], such as ventilatory O_2_ convection. Previous studies indicated that the increased hypoxia would cause an increase in ventilation and breathing frequency in amphibian and reptilian [25,26]. Alternatively, having large lungs also can increasing O_2_ diffusion as it increases the surface area where diffusion occurs. Our results indicated that lung mass also increased with increasing altitude in *P*. *vlangalii*, suggesting this species might compensate hypoxia by increasing ventilatory O_2_ convection. However, a significant positive effect of air pressure on lung mass was detected using GLMM, indicating further studies need to be performed.

Altitude exposure also induces major changes in cardiovascular function, such as tachycardia, pulmonary hypertension [7,9], and heart mass [6,27], which can increase cardiac output. This plasticity mainly concerns the initial physiological responses to high altitude, and most of these changes actually correspond to maladaptation [5]. Our results suggest that during ancestral acclimatization, blood O_2_ transport might not have been compensated by a cardiac output increase in *P*. *vlangalii*, as heart mass did not change with altitude. However, GLMM results indicated that hypoxia had an positive effect on heart mass, and this species might have, therefore, compensated for the low O_2_ transportation increasing hemoglobin concentration, hematocrit, and hemoglobin binding-affinity to O_2_ [5]. This is supported by recent studies comparing *Phrynocephalus* spp. populations, which revealed that those inhabiting high altitudes had higher hematocrit, hemoglobin concentration, and oxygen carrying capacity than those inhabiting low altitudes [6,11,27,28].

Low temperature is another environmental pressure affecting animals living at high altitude, especially ectotherms. To acclimate to cold, *Phrynocephalus* spp. lizards inhabiting high altitudes evolved a lower optimal body temperature and critical thermal minimum than lizards inhabiting low altitudes [29]. In order to overcome the disadvantage of developing embryos in low temperatures, these species also evolved viviparity [30] and an atypical reproductive cycle [31]. In our study, we found that low temperature also affected heart, lung, and intestinal track mass in *P*. *vlangalii*.

For ectotherms, ventilation is normally regulated to meet the needs for CO_2_ elimination, to support the increased metabolic rate with increasing temperature [32]. *Phrynocephalus* spp. always decrease their optimal body temperature in response to cold [29], resulting in a standard metabolic rate decrease [6], which would reduce their pulmonary ventilation as expected in ectotherms. However, lung mass increased with decreasing temperature in *P*. *vlangalii*, against the above hypothesis. Pulmonary ventilation could be changed through tidal volume, breathing rate or both. Unfortunately, our samples did not allow estimating any of these, although we tried to verify tidal volume increased in *P*. *vlangalii* with decreasing temperature. Thus, further research is needed on *P*. *vlangalii* pulmonary ventilation response to cold.

Under low temperatures, small mammals’ heat-loss increases resulting in increasing energy demand and intake, which lead to changes in the mass of small intestine and heart [3]. Our results indicated that ectotherms living in cold conditions also have a relatively larger heart and intestinal track than those living in warm environments. However, as mentioned above, ectotherms always suppress metabolism in response to low temperature and thus the larger organ mass found might be due to an increase in energy intake. A rigorous study on *P*. *vlangalii’s* energy budget and food intake is therefore needed to enable a clear interpretation of the relationships between decreasing temperature and organ mass increase.

## Supporting Information

S1 Table Sample ID for 138 *Phrynocephalus vlangalii* used in this study. ^a^ samples which only collected heart and lung mass. ^b^ samples which only collected stomach and intestinal tract mass.
(DOCX)
