# Supplementary material for: Organ Mass Variation in a Toad Headed Lizard Phrynocephalus vlangalii in Response to Hypoxia and Low Temperature in the Qinghai-Tibet Plateau, China
Source: PLoS One. 2016 Sep 7;11(9):e0162572. doi: 10.1371/journal.pone.0162572 (PMC5015776; doi:10.1371/journal.pone.0162572)
Supplement: S1 Table — a samples which only collected heart and lung mass. b samples which only collected stomach and intestinal tract mass. (DOCX) [file pone.0162572.s001.docx]

**S1 Table. Sample ID for 138 *Phrynocephalus vlangalii* used in this study.**

| Population | Specimen sample number |
| --- | --- |
| Golmud(19) | 14005, 14007, 14008, 14009, 14010, 14012, 14015, 14035, 14037, 14038, 14039, 14040, 14041, 14044, 14047, 14048, 14049, 14051, 14053 |
| Delingha(46) | DLH12a-1^a^, DLH12a-9^a^, DLH12a-10^a^, DLH12a-12^a^, DLH12a-13^a^, DLH12a-17^a^, DLH12a-21^a^, DLH12a-27^a^, DLH12a-31, DLH12a-32, DLH12a-33, DLH12a-37, DLH12a-38, DLH12a-36^a^, DLH5^a^, DLH12^a^, DLH18^a^, DLH23^a^, DLH35^a^, DLH39^a^, DLH40^a^, 14063^a^, 14064^a^, DLH28^b^, DLH3, DLH7, DLH11, DLH14, DLH17, DLH24, DLH25, DLH26, DLH27, DLH31, DLH32, DLH33, DLH41, DLH48, DLH52, DLH54, DLH60, DLH64, DLH67, 14058, 14060, 14062 |
| Daotanghe(31) | DTH12a-4, DTH12a-10^a^, DTH12a-12, DTH12a-13^a^, DTH12a-14^a^, DTH12a-15^a^, DTH12a-24^a^, DTH12a-25^a^, DTH12a-26, DTH12a-27^a^, DTH12a-29^a^, DTH12a-32^a^, DTH12a-33^a^, DTH12a-35^a^, DTH12a-37^a^, DTH15^a^, DTH21^a^, DTH23, DTH26, DTH30^a^, DTH31, DTH32^a^, DTH36^a^, DTH37, DTH38^b^, DTH39^a^, DTH42, DTH45^b^, DTH98^a^, DTH102^a^, DTH103 |
| Maduo(42) | MD12a-1, MD12a-3^a^, MD12a-10, MD12a-11, MD12a-12, MD12a-13, MD12a-14, MD12a-16, MD12a-26, MD12a-27, MD12a-31, MD12a-35, MD12a-37, MD12a-39, MD12a-41^a^, MD12a-42, MD7, MD14^a^, MD15^a^, MD23, MD24, MD26, MD28, MD30, MD31, MD32^a^, MD33, MD34, MD35, MD37, MD38, MD77, MD83^a^, MD152^a^, MD163^a^, MD168^a^, MD170^a^, MD174^a^, MD176^a^, MD178^a^, MD179^a^, MD180^a^ |

^a^ samples which only collected heart and lung mass.

^b^ samples which only collected stomach and intestinal tract mass.
